# Three unreported cases of TMEM199-CDG, a rare genetic liver disease with abnormal glycosylation

**DOI:** 10.1186/s13023-017-0757-3

**Published:** 2018-01-10

**Authors:** Pietro Vajro, Katarzyna Zielinska, Bobby G. Ng, Marco Maccarana, Per Bengtson, Marco Poeta, Claudia Mandato, Elisa D’Acunto, Hudson H. Freeze, Erik A. Eklund

**Affiliations:** 1 Unit of Pediatrics, Department of Medicine, Surgery and Dentistry, Scuola Medica Salernitana, University of Salerno, Baronissi, (Sa) Italy; 2 Division of Pediatrics, Lund University, Lund, Sweden; 3 Human Genetics Program, Sanford Burnham Prebys Medical Discovery Institute, La Jolla, California USA; 4 Section for Matrix Biology, Department of Experimental Medical Sciences, Lund University, Lund, Sweden; 5 Division of Clinical Chemistry, Department of Clinical Sciences, Lund University, Lund, Sweden; 6 Children’s Hospital “Santobono-Pausilipon”, 1st Division of Pediatrics, Naples, Italy

**Keywords:** CDG, Glycosylation, Ceruloplasmin, Transferrin, Liver disease, Transaminase, TMEM199

## Abstract

**Background:**

*TMEM199* deficiency was recently shown in four patients to cause liver disease with steatosis, elevated serum transaminases, cholesterol and alkaline phosphatase and abnormal protein glycosylation. There is no information on the long-term outcome in this disorder.

**Results:**

We here present three novel patients with TMEM199-CDG. All three patients carried the same set of mutations (c.13-14delTT (p.Ser4Serfs*30) and c.92G > C (p.Arg31Pro), despite only two were related (siblings). One mutation (c.92G > C) was described previously whereas the other was deemed pathogenic due to its early frameshift. Western Blot analysis confirmed a reduced level of TMEM199 protein in patient fibroblasts and all patients showed a similar glycosylation defect. The patients presented with a very similar clinical and biochemical phenotype to the initial publication, confirming that TMEM199-CDG is a non-encephalopathic liver disorder. Two of the patients were clinically assessed over two decades without deterioration.

**Conclusion:**

A rising number of disorders affecting Golgi homeostasis have been published over the last few years. A hallmark finding is deficiency in protein glycosylation, both in N- and O-linked types. Most of these disorders have signs of both liver and brain involvement. However, the present and the four previously reported patients do not show encephalopathy but a chronic, non-progressive (over decades) liver disease with hypertransaminasemia and steatosis. This information is crucial for the patient/families and clinician at diagnosis, as it distinguishes it from other Golgi homeostasis disorders, in having a much more favorable course.

## Background

The differential diagnoses of elevated transaminases in children include a large variety of infectious, autoimmune, metabolic, genetic, gastrointestinal, and extrahepatic disorders [1]. Recently a novel disease with chronically elevated serum transaminases and low serum ceruloplasmin, caused by mutations in the gene encoding the transmembrane protein TMEM199 [2]. These patients also showed deficient transferrin (TF) glycosylation with a Type 2 congenital disorder of glycosylation (CDG) pattern. TMEM199 is the human orthologue of yeast Vma12p, which, together with Vma21p and Vma22p, constitute a complex that chaperones the assembly of the V_0_ domain of the Vacuolar H+ ATPase (V-ATPase) [3]. This proton pump is responsible for the acidification of the endosomes of the secretory pathway, including the lysosome and the Golgi apparatus. Failure to acidify the Golgi apparatus may affect the complex Golgi-located glycosylation machinery, leading to a glycosylation defect [4]. A decade ago we published a report on four children with glycosylation deficiencies with liver disease [5], but without known genetic cause. Two of these children were later diagnosed with phosphoglucomutase-1 CDG (PGM1-CDG; OMIM 614921) [6]. In this report, we present a long-term follow-up on the other two children, and present data on a third, unrelated child, all diagnosed with TMEM199-CDG (OMIM 616829).

## Methods

### Glycosylation studies

The LC-MS of TF was done as previously described [7].

### Molecular analysis of TMEM199

Mutation analysis for *TMEM199* (NM_152464.2, ENST00000292114) was performed by direct gene sequencing of all six coding exons, including the exon-intron boundaries.

### Western blot analysis

Patient and control skin fibroblasts were set up and grown as previously described [8]. HepG2 cells were purchased from ATCC (Manassas, VA) and maintained as suggested. 3 × PBS-washed cell layers were lysed in RIPA buffer (50 mM TrisHCl, pH 7.4, 150 mM NaCl, 1% Triton X-100, 0.5% Sodium deoxycholate, 0.1% SDS, and 1 mM EDTA) containing protease inhibitor tablets (cOmplete™ Mini, Roche) and 8 μg of protein per lane each were run on stain-free BioRad gels and blotted to PVDF membranes. These were blocked using 5% BSA in TBST and incubated with the primary anti-TMEM199 antibody (Abnova PAB21999, Abnova, Taiwan; 0,5 μg/mL) over night, followed by incubation with a goat anti-rabbit HRP-conjugated antibody (ab6721, Abcam) and the ECL Plus Western Blotting Detection Reagent (GE Healthcare Life sciences). The blots were developed using the imaging system SuperSignal West Dura (Thermo Scientific) Loading control are bands detected on the membrane using ChemiDoc Touch Imaging System BioRad.

## Results

The patients/their legal guardians gave informed consent for this study.

**Fig. 1:**
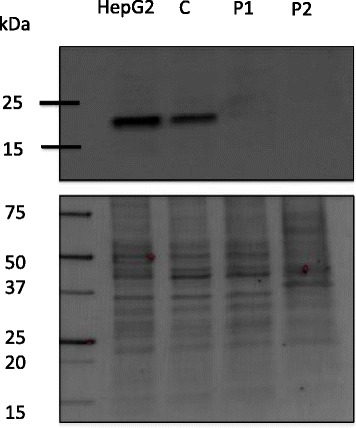
Western Blot analysis of TMEM199 protein. Cell layer protein extracts from patient 1 (P1) and patient 2 (P2) fibroblasts were probed for TMEM199 using anti-TMEM199 antibodies (upper gel). An in-house control fibroblast cell line (C) and the commercially available HepG2 hepatocyte cell line were used as controls. The expected position of TMEM199 is at around 19 kDa. Bands detected on the membrane (lower gel) were used as loading controls

Patient 1 and patient 2 (siblings) are healthy young adults with normal physical and psychological development. They were investigated for hypertransaminasemia and slight liver enlargement at 2 years of age. They also presented with increased values of total and LDL serum cholesterol, creatine kinase (CK), and alkaline phosphatase (ALP). Serum ceruloplasmin and serum copper were low in spite of a normal urinary excretion of copper at basal level and after penicillamine. Liver histology showed mild periportal fibrosis and focal steatosis. The liver copper content was mildly increased, but molecular studies ruled out Wilson’s disease [9]. Serum, liver and brain (MRI) did not show increased metal storage, thus ruling out aceruloplasminemia. Serum TF glycosylation analysis was consistent with a Type 2 CDG [5]. At a recent follow-up (at age 27 and 24 years, respectively) both patients were clinically well, and there was no sign of progressive liver, bone or neuromuscular disease, nor any signs of malignant tumors. Exome-sequencing was performed identifying compound heterozygous mutations in *TMEM199* (c.13-14delTT, c.92G > C), causing a frameshift with a premature stop (p.Ser4Serfs*30) and a missense change (p.Arg31Pro), respectively. The Genome Aggregation Database (gnomAD) (http://gnomad.broadinstitute.org/) (10.19.2017 ver2.0) [10] of 123,136 exome and 15,496 whole-genome sequences contains 10 heterozygotes from 121,705 apparently healthy individuals (0 homozygotes) for the p.Arg31Pro exchange, but no alleles carrying the c.13-14delTT, yielding a carrier frequency of 0.000082 and 0, respectively). Western Blot analysis could not detect the TMEM199 protein in patient fibroblasts, but showed a strong signal in both control fibroblasts and in HepG2 control hepatocytes (Fig. 1).

**Fig. 2:**
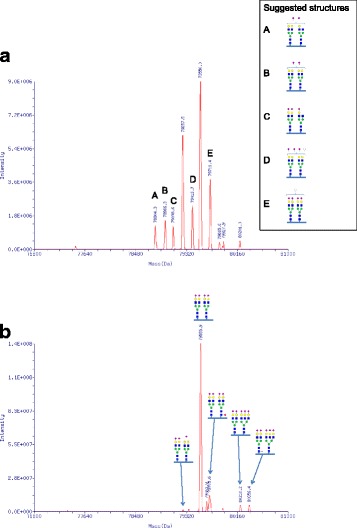
Mass spectrometric analysis of transferrin (TF). Patient 3 (**a**) and control (**b**) sera were analyzed using LC-MS analysis. Deconvoluted masses of intact serum TF from full scans are shown. TF masses representing confirmed glycoconjugate structures are indicated with respective schematic glycoconjugate structures (blue squares, N-acetylglucosamine; green circles, mannose; yellow circles, galactose; pink diamonds, sialic acid (NeuAc); red triangles, fucose; white circles, hexose) where deviating masses are indicated with letters and suggested structures are shown beside

Patient 3 is a clinically healthy 2-year-old child who, at 10 months of age, was diagnosed with mild hypertransaminasemia as an incidental finding. Liver ultrasonography revealed a mild steatosis and the follow-up showed fluctuating values of serum transaminases, and elevated ALP and CK. Other liver tests were normal except antithrombin-III activity of 68% and low ceruloplasmin (8 mg/dL, normal range 20–46). Mass spectrometric analysis of serum TF glycosylation showed a pathological pattern (CDG-II, Fig. 2a and b) similar to Patient 1 and Patient 2 [5]. We therefore performed direct Sanger sequencing of *TMEM199* for Patient 3 and found the same set of compound heterozygous mutations as seen in Patient 1 and Patient 2. The two families have no known common ancestors, but both originate from the same region of Campania, Southern Italy. Table 1 summarizes clinical and laboratory features of the three patients.

**Table 1:** Clinical and laboratory findings in the 3 patients with TMEM199-CDG

	Family 1				Family 2	
	Patient 1		Patient 2		Patient 3	
	Onset	Last follow-up	Onset	Last follow-up	Onset	Last follow-up
Age (years)	2	27	2	24	1.4	2.4
Gender	F		M		M	
Glycosylation profile	Type 2 CDG pattern		Type 2 CDG pattern		Type 2 CDG pattern	
Symptoms	None	None	None	None	None	None
Clinical Examination	Slight hepatomegaly	normal	Slight hepatomegaly	normal	Slight hepatomegaly	normal
Neurological Development	Normal	Normal	Normal	Normal	Mild Delay of Speech	Normal
Brain MRI and mineral content	Normal		Normal		Not done	
Malformations	None		None		None	
AST (nv < 41 U/L)	349	53	299	98	656	156
ALT (nv < 45 U/L)	329	23	221	50	437	104
ALP (nv < 475 U/L)	1995	1140	3990	903	1235	713
TOTAL CHOL (nv < 200 mg/dL)	340	300	240	220	140	160
HDL CHOL (nv > 45 mg/dL)	49	54	45	46	49	54
LDL-CHOL (nv < 160 mg/dL)	256	240	176	177	96	98
CK (nv 0-170 U/L)	799	561	442	1428	510	204
Ceruloplasmin (nv 20-46 mg/dL)	6	8	4	6	8	8.4
Haptoglobin (nv 30-250 mg/dL)	20	40	40	40	30	30
Serum Copper (nv 69-122 μg/dL)	< 40	< 40	< 40	< 40	< 40	< 40
Urinary Copper (basal and after penicillamine)	Normal	Normal	Normal	Normal	Not Done	Normal
Coagulation Parameters	Normal/Borderline	Normal	Normal/Border-line	Normal	Normal	Low ATIII activity
Liver Ultrasonography	Bright liver	Inhomogeneous echogenicity	Bright liver	Normal	Hepatomegaly Bright liver	Hepatomegaly Bright liver
Liver Histology	Mild periportal fibrosis; focal steatosis (ages 6 & 9)		Mild periportal fibrosis; focal steatosis (ages 2 & 5)		Not done	
Liver EM^a^	No Wilsonian changes (age 6)		No Wilsonian changes (age 6)		Not done	
Liver Copper (nv < 50 μg/g dry weight)	318 μg/g at age 6;280 μg/g at age 9		250 μg/g at age 2: 312 μg/g at age 5		Not done	
Wilson Disease molecular study^b^	Negative		Negative		Negative	
Treatments	Vitamin D × 1 yr at age 4; Penicillamine for 6 mos at age 5 with no effects		None		None	

*Abbreviations*: *ALP* alkaline phosphatase, *ALT* alanine aminotransferase, *AST* aspartate aminotransferase, *AT III* antithrombin III, *CHOL* cholesterol, *CK*, creatine kinase, *EM* electron microscopy, *MRI* magnetic resonance imaging

^a^Courtesy of Prof. I. Sternlieb; NY, USA

^b^Courtesy of Dr. J Loudianos, University of Cagliari – Italy

## Discussion

The causes of elevated transaminases are many, spanning from transient, harmless conditions to ones associated with a progressive, life-threatening course [1]. Here we present three patients with TMEM199-CDG, a recently described genetic cause of hypertransaminasemia [2]. They all carried the same set of compound heterozygote mutations, despite no known common family ancestors. One of the mutations, c.92G > C, was previously reported by Jansen et al. [2], whereas the other one is pathogenic due to its early frameshift/termination. Using Western Blot analysis in patient fibroblasts, it was shown that there is no measurable amount of TMEM199 protein, indicating that the missense mutation causes a diminished production or a rapid degradation of the resulting protein. A protein product from the c.13-14delTT allele would not be picked up by the antibody used in the Western Blot, however, given its early termination, we think it is very unlikely that such a protein would be functional at al. The clinical and biochemical findings in our patients were consistent with the previously published cases, and included, apart from hypertransaminasemia, deficient TF glycosylation and low ceruloplasmin, slightly increased copper content of the liver, mild non-progressive liver fibrosis and normal (except one patient [2]) psychomotor development. The only patient that has been diagnosed with symptoms from the nervous system, including benign hypotonia and psychomotor developmental issues [2], has a homozygous mutation (c.92G > C) in *TMEM199*, which could indicate consanguinity and thus a possible second mutation as a cause of these symptoms. Notably, the clinical course for two of these patients has been stable over two decades, with no deterioration of the liver pathology or development of other symptoms. TMEM199 is the human orthologue of yeast Vma12p, which acts as a chaperone in the formation of the V-ATPase, the proton pump responsible for the acidification of the vesicles of the secretory pathway [3]. Why TMEM199-CDG mainly presents with hepatopathy is enigmatic. Mutations in a number of genes involved in the formation of the V-ATPase (either encoding subunits or assembly factors) cause a plethora of symptoms with either systemic or organ specific features [11–15]. In several of these disorders, pathological TF glycosylation (Type 2 patterns) is detected, as in patients with mutations in *ATP6V0A2* (OMIM 219200) [14], *ATP6V1A* (OMIM 617403) and *ATP6V1E1* (OMIM 617402) [15], *ATP6AP1* (OMIM 300972) [13] and *CCDC115* (OMIM 616828) [11]. This was also the case in our patients as well as in the four previously published TMEM199-CDG patients [2]. The glycosylation pattern indicates a Golgi related problem (CDG-II) stressing the need for a tight pH regulation in the Golgi stacks for proper function of the glycosylation enzymes. Disturbances in copper metabolism in patients with CDG-II [16] as well as in V-ATPase deficiencies [11, 13, 15] have been reported. These include hepatopathy with low serum ceruloplasmin and low serum copper. In both TMEM199-CDG and CCDC115-CDG, a modest accumulation of copper in the liver is also seen (this report and [11]. The mechanisms behind these disturbances are at the moment unclear, but potentially involve at least partial loss of either or both of the copper transporting proteins ATP7A and ATP7B. In Wilson’s disease (ATP7B deficiency) [17], export of copper from the hepatocyte (and neuron) is deficient. This causes low serum ceruloplasmin, low serum copper but accumulation of copper in the liver, similar to the TMEM199-/CCDC115-CDG patients. However, ATP7B is not glycosylated [18] and its transfer of copper is not pH dependent [19], why a potential link between V-ATPase malfunction and loss of ATP7B mediated copper transportation might thus reside in the synthesis or degradation of the ATP7B protein. This has not yet been investigated. In the case of ATP7A related disorders (Menkes disease or occipital horn syndrome), the uptake of intestinal copper is deficient, leading to low serum copper, low ceruloplasmin but also low copper in liver tissue [20]. ATP7A is, in contrast to ATP7B, glycosylated [18] and deficient glycosylation could potentially affect its function. Also, a very recent study showed that several COG subunits (involved in Golgi homeostasis and glycosylation) are within the interactome of ATP7B, linking Golgi homeostasis, glycosylation and intracellular copper homeostasis [21]. The patients with TMEM199-CDG described so far have displayed a strikingly similar clinical presentation, with hepatic features resembling other V-ATPase chaperone deficiency syndromes, however without associated symptoms such as hypogammaglobulinemia [13], epilepsy and cognitive impairment [11, 13] noted in the related syndromes.

Interestingly, it was recently published that loss of TMEM199 (and CCDC115) rescues the angiogenic factor HIF-1α from proteasome-mediated degradation under normoxic conditions by intracellular iron depletion [3]. If this is true also in man, it may cause a chronically elevated HIF-1α activity in our patients, potentially increasing their risk of developing cancer. However, in the present adult patients, no suspicion of malignancy has yet occurred.

## Conclusions

Three novel TMEM199-CDG cases show a biochemical and clinical phenotype in accordance with the previously described patients. In our patients, the disease course seems stable and mild over several decades, why it is possible that an early diagnosis of TMEM199-CDG may allow for the avoidance of patient and parental worries and unnecessary repeat blood sampling. There is, however, a need for more patient descriptions of this rare condition to better understand the disease course and further elucidate whether TMEM199-CDG will remain a liver disease only.

## Abbreviations

ALP: Alkaline phosphatase
CDG: Congenital disorder of glycosylation
CK: Creatine kinase
TF: Transferrin
V-ATPase: Vacuolar H+ ATPase
